# Ovarian metastasis of Müllerian adenosarcoma of the cervix with sarcomatous overgrowth

**DOI:** 10.4274/tjod.75735

**Published:** 2017-09-30

**Authors:** Meral Koyuncuoğlu, Bahadır Saatli, Nuri Yıldırım

**Affiliations:** 1 Dokuz Eylül University Faculty of Medicine, Department of Pathology, İzmir, Turkey; 2 Dokuz Eylül University Faculty of Medicine, Department of Obstetrics and Gynecology, İzmir, Turkey; 3 Ege University Faculty of Medicine, Department of Obstetrics and Gynecology, İzmir, Turkey

**Keywords:** Cervix, Müllerian adenosarcoma, ovarian metastasis

## Abstract

The aim of this study is to present a rare case of Müllerian adenosarcoma of the cervix with ovarian metastasis and sarcomatous overgrowth. A gravida 2, para 2 woman aged 32 years with vaginal bleeding was admitted to the gynecology department. A 3-4 cm polypoid mass protruding from the cervix was detected in a pelvic examination. Total abdominal hysterectomy and bilateral salpingoopherectomy was performed because of metastatic implants on the right ovary. The pathologic evaluation revealed Müllerian adenosarcoma of the cervix with sarcomatous overgrowth and ovarian metastasis. After surgery, the patient was planned to undergo chemo- and radiotherapy. This is the first cervical Müllerian adenosarcoma case mentioned in the literature with metastasis to the ovary in a young woman. There is no optimal management option for cervical adenosarcomas due to the rarity of this phenomenon. Nevertheless, even if the patient is young and imaging techniques do not elucidate metastatic disease, surgeons should evaluate the ovaries for the spread of tumor, especially if histology reveals sarcomatous overgrowth.

## INTRODUCTION

Müllerian adenosarcoma (MA) is relatively a rare type of mixed epithelial and mesenchymal tumor of the uterus. It is usually composed of a benign epithelial component and a low-grade stromal-sarcomatous component. Occasionally, the epithelial component may be atypical and sarcomatous overgrowth may be seen in the stromal component^(1,2,3)^. MA mostly occurs in the uterus but also in extra-uterine sites, particularly in the ovary.

The tumors generally originate from the uterine corpus/endometrium, and less frequently from the uterine cervix. Adenosarcoma usually presents with abnormal vaginal bleeding. If the tumor originates from the cervix, the tumor is commonly seen as a polypoid tissue protruding from the external cervical ostium like a cervical polyp on gross evaluation^(3,4)^. Cervical adenosarcoma, especially with sarcomatous overgrowth, is extremely rare. Distant metastasis is also extremely rare because the epithelial component is usually benign and the stromal component is generally low-grade^(1)^. In this report, we present a MA originating from the endocervical canal with sarcomatous overgrowth including heterologous elements and ovarian metastasis in a young woman.

## CASE REPORT

A gravida 2, para 2 woman aged 32 years with vaginal bleeding was admitted to the gynecology department. She reported having abnormal vaginal bleeding for a couple of years and a benign endometrial biopsy in another gynecology center 6 months ago. The pelvic examination revealed a 3-4 cm polypoid mass protruding from the cervix to the vagina. The uterus and the ovaries were normal in size. Transvaginal sonography revealed an irregular-shaped polypoid structure lying from the internal cervical ostium level to the vagina whose thickness was 16 mm. Magnetic resonance imaging (MRI) revealed a lesion starting from the endocervical canal lying to the vagina and there were no pathologic lymph nodes. Her serum CA125 and CA19-9 levels were within normal limits. A biopsy from the polypoid lesion revealed MA with sarcomatous overgrowth.

**Figure 1:**
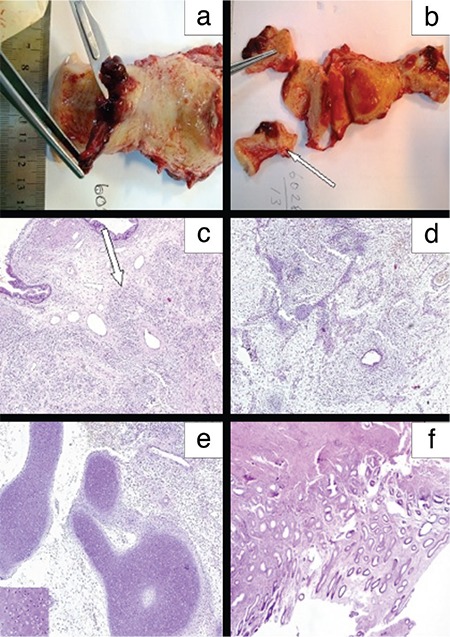
The tumor was seen as polyps (a) and invaded the outer third of the myometrium macroscopically (b). The tumor originated from the cervix, and sarcomatous component was over 25% (c, d). Large foci of benign cartilage (inset) were seen as heterologous element (e). The non-tumoral endometrium was seen (f)

During surgery, metastatic implants were observed over the right ovary and the left ovary was solid in appearance. The outer surface of the uterus was normal. Total abdominal hysterectomy and bilateral salpingoopherectomy was performed. Three polyps were seen in the endocervical canal, which measured between 0.6x0.5x0.3 and 2x0.8x0.5 cm. The cut surfaces of the polyps were solid and grey-white in appearance. Hemorrhage was seen because of previous biopsy. The tumor had invaded the outer half of the myometrium macroscopically (Figure 1a, 1b).

**Figure 2:**
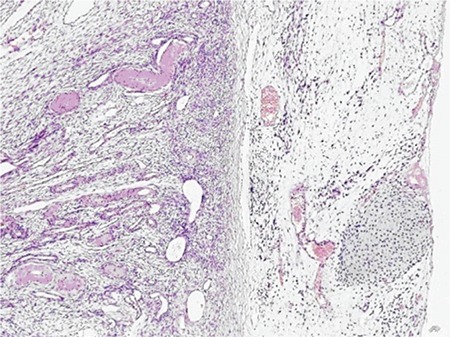
The ovarian metastasis showed sarcomatous component with myxoid background and foci of benign cartilage

The final report confirmed that the tumor originated from the endocervical canal. It invaded the lower uterine segment and the myometrium, less than the outer half, and consisted of elongated spindle cells with a myxoid background and few benign glandular formations. The sarcomatous stroma made up 80% of the tumor. In these areas, paucicellular and hypercellular morphology with myxoid stroma were seen. Low-grade and high-grade stromal components were mixed. Mitotic activity was 4 per 10 high-power fields (HPFs) and 2 per 10 HPFs in paucicellular and hypercellular areas, respectively. Immunohistochemistry showed positive immunoreactivity with vimentin and desmin, and negative immunoreactivity to actin, s-100, and CD99. The glandular epithelium was primarily of endocervical type without atypia and mitotic activity. Large foci of benign cartilage were seen in the sarcomatous areas (Figure 1c, 1d, 1e). There was no endometrial involvement (Figure 1f). The tumor invaded the ovaries and there were no lymphovascular space invasion. The ovarian metastasis showed the same morphology (Figure 2). Intraoperative peritoneal lavage cytology was positive for malignant cells. The patient was discussed in the gynecologic oncology board and at the present time she has been taking a chemotherapy regimen of ifosfamide and doxorubicin. Then she is planned to have both pelvic and vault radiotherapy.

## DISCUSSION

MA is an infrequent type of sarcoma of the female genital system. It mostly comprises a low-grade malignant stromal component and benign epithelial component. It commonly arises from the uterine corpus/endometrium. Cervical adenosarcoma is seen in 2-9% of all MAs^(5,6,7,8)^. The age at clinical presentation in cervical adenosarcoma is lower than that with uterine corpus, which is about 27 years^(3,4,5,6)^. This is concordant with our patient whose age was 32 years. Chin et al.^(9)^ also showed that 89% of patients were premenopausal. The most frequent symptom is abnormal vaginal bleeding, as in our case^(9)^. Generally, the most common finding in physical examination is cervical polypoid lesion protruding from external cervical ostium to the vagina. The diagnosis can be made through biopsy from the lesion.

Sarcomatous overgrowth is defined as overgrowth of the neoplasm by a pure sarcomatous component occupying at least 25% of the lesion^(1)^. This subtype has a more malignant behavior than the classic adenosarcoma^(10)^. The rate of myometrial invasion and extension to the serosal surface, recurrence rate, and death because of tumor progression are all higher in adenosarcomas with sarcomatous overgrowth^(10,11)^.

Heterologous elements are rarely reported, accounting for 8-42% of cervical adenosarcoma^(3,4)^. Mostly, these elements are cartilage or striated muscle. Charfi et al.^(12)^ also reported a patient with adenosarcoma of the cervix with sarcomatous overgrowth and heterologous elements that contained cartilage and presented as a recurrent cervical polyp.

Cervical adenosarcomas with sarcomatous overgrowth in young patients are very rare. In particular, ovarian metastasis at the time of diagnosis is almost not mentioned in the literature. A recent study by Tanner et al.^(13)^ reported that ovarian metastases were not identified in any patients undergoing bilateral salpingoopherectomy in 16 patients with MA. The present case is one of the first with cervical adenosarcoma metastases to the ovary.

The optimal therapy and the prognostic factors for these are still unclear because the reports of these cases are insufficient and long-term follow-up data are absent. The extensiveness of surgery may differ from patient to patient. Chin et al.^(9)^, reported 9 cases of cervical adenosarcoma. They performed a cervical wedge resection for a patient who desired to preserve fertility. Hysterectomy, bilateral salpingoopherectomy, and lymphadenectomy were performed for other patients, none of whom had sarcomatous overgrowth. Park et al.^(14)^ performed hysterectomy, bilateral salpingoopherectomy, and lymphadenectomy for a 37-year-old woman with adenosarcoma of the cervix with sarcomatous overgrowth. However, there was no tumor in the ovaries and no lymph node metastasis^(14)^. Manoharan et al.^(15)^ reported three patients with cervical adenosarcoma. The first one was a 28-year-old woman with sarcomatous overgrowth. She underwent radical hysterectomy with preservation of the ovaries. The histology revealed lymphovascular space and right parametrial invasion, so she had chemotherapy followed by both pelvic radiotherapy and brachytherapy. The second case was a 26-year-old woman who had cervical MA with spindle cell stroma and benign glands. Vaginal hysterectomy with preservation of the ovaries and laparoscopic lymph node dissection was performed. The lymph nodes were free of tumor. She was given adjuvant pelvic radiotherapy because of deep stromal infiltration and high-grade tumor. The third case was a 41-year-old woman who had cervical adenosarcoma with mild-to-moderate stromal cytological atypia and underwent abdominal hysterectomy with preservation of ovaries. She received no adjuvant therapy because the tumor was localized to the polyp. Our patient was also a young woman and we were planning to preserve the ovaries before surgery. However, we came across the metastatic implants on the right ovary intraoperatively. MRI and ultrasonography did not facilitate the management preoperatively.

As previously mentioned, this is a very rare case and there is no consensus on either surgical or adjuvant treatment strategy. The first point that physicians should keep in mind is the number of mitotic figures in the mesenchymal part because the epithelial part is benign. Secondly, sarcomatous overgrowth may determine the behavior of the disease, as it was in our patient. If there is sarcomatous overgrowth, surgeons should carefully evaluate the patient for distant or locoregional spread.

As a result, there is no optimal management option for cervical adenosarcomas due to the rarity of this phenomenon. Nevertheless, even if the patient is young and imaging techniques fail to reveal metastatic disease, surgeons should evaluate the ovaries for the spread of tumor, especially if the histology reveals sarcomatous overgrowth.
